# Experimental Observation of the High Pressure Induced Substitutional Solid Solution and Phase Transformation in Sb_2_S_3_

**DOI:** 10.1038/s41598-018-33035-4

**Published:** 2018-10-04

**Authors:** Yingying Wang, Yanmei Ma, Guangtao Liu, Jianyun Wang, Yue Li, Quan Li, Jian Zhang, Yanming Ma, Guangtian Zou

**Affiliations:** 1 College of Physics, State Key Laboratory of Superhard Materials, Jilin University, Changchun, 130012 China; 2 National Key Laboratory of Shock Wave and Detonation Physics, Institute of Fluid Physics, Chinese Academy of Engineering Physics, Mianyang, 621900 China

## Abstract

The substitutional solid solutions composed of group VA-VIA nonmetallic elements has attracted considerable scientific interest since they provide a pressure-induced route to search for novel types of solid solutions with potential applications. Yet, the pressure-induced solid solution phase is unprecedented in the sulfide family. In this paper, the structural behavior of antimony trisulfide, Sb_2_S_3_, has been investigated in order to testify whether or not it can also be driven into the substitutional solid solution phase by high pressures. The experiments were carried out by using a diamond anvil cell and angle dispersive synchrotron X-ray diffraction up to 50.2 GPa at room temperature. The experimental results indicate that Sb_2_S_3_ undergoes a series of phase transitions at 5.0, 12.6, 16.9, 21.3, and 28.2 GPa, and develops ultimately into an Sb-S substitutional solid solution, which adopts a body-centered cubic disordered structure. In this structure, the Sb and S atoms are distributed randomly on the *bcc* lattice sites with space group *Im-3m*. The structural behavior of Sb_2_S_3_ is tentatively assigned by comparison within the A_2_B_3_ (A = Sb, Bi; B = Se, Te, S) series under high pressures.

## Introduction

Alloys, especially substitutional solid solutions, have been widely used in modern engineering and industry due to their superior physicochemical properties and performances than the corresponding constituent end members. The exploration for novel alloys is one of the major goals of the scientific community. Substitutional binary solid solutions are usually composed of metallic elements, such as Mn-Cu, Au-Ag, Ni-Cr, etc. The solid solutions made up of nonmetallic elements are rarely reported until a solid solution for Bi-Te binary system has been established under high pressures^1,2^. Bismuth telluride, Bi_2_Te_3_, a stoichiometric semiconductor, develops unexpectedly into a Bi-Te substitutional solid solution through pressure-induced structural phase transition. The high-pressure substitutional solid solution phase, denoted as phase IV, appears above 15.5 GPa and remains stable at least up to 52.1 GPa. The structural model of phase IV may be built up with a body-centered cubic lattice with space group *Im-3m* with the Bi and Te atoms distributed randomly on the lattice sites. Later on, the low-period counterparts of Bi_2_Te_3_ in the periodic table, Bi_2_Se_3_, Sb_2_Te_3_, and Sb_2_Se_3_, also exhibit the tendency to turn into substitutional solid solutions under high pressures. The Sb-Te substitutional solid solution with the bcc disordered structure can be obtained when Sb_2_Te_3_ is compressed at 21.6 GPa and higher pressures^3^. The high pressure X-ray diffraction (XRD) patterns of this cubic phase strictly follows the extinction rules for space group *Im-3m* and allows only one inequivalent atomic position at 2a (0, 0, 0). Above 51 GPa, new Bragg peaks appear in the XRD patterns of Sb_2_Se_3_, indicative of a pressure-induced structural transition and the high-pressure phase can be assigned to the simple, yet disordered, bcc structure, which forms the Sb-Se substitutional solid solution^4^. The situation is somehow complicated as for the case of Bi_2_Se_3_. Upon compression at pressures above 22.1 GPa, Bi_2_Se_3_ develops into a novel phase with a complex XRD pattern that is tentatively assigned to a monoclinic C2/m structure with 9/10-fold coordination^5^. Note that the 9/10-fold C2/m structure does have a bcc-like local order and thus show great similarity in the overall theoretical and experimental XRD patterns^1^. However, the systematic extinction rules of neither the 9/10-fold C2/m structure nor the bcc disordered structure can be unambiguously applied to the complex experimental XRD patterns. Hence the probability of the transformation of Bi_2_Se_3_ into the substitutional solid solution phase cannot be ruled out completely. Then comes the question that whether or not the sulfides of these group VA elements, A_2_S_3_ (A = Sb and Bi), can also be driven into the substitutional solid solution phase by high pressures. It is reported that the ambient-pressure *Pnma* structure of Bi_2_S_3_ is found to persist up to 50 GPa through a combination of experimental and theoretical studies^6^. Further compression leads to structural disorder and amorphization. However, in the case of Sb_2_S_3_, contradictions exist in the reported results in the literature, which needs to be testified by further experimental and theoretical studies^7,8^. Nevertheless, to the best of our knowledge, the pressure-induced substitutional solid solution phase is unprecedented in the sulfide family of these group VA elements.

The interests for the high pressure studies on these group VA sulfides A_2_S_3_ (A = Sb and Bi) also arises from the expectations that structural modulations and properties enhancements may be achieved by applying external pressure. First, the investigation of the high-pressure properties of A_2_S_3_ and related compounds is particularly relevant for the search of possible topological states, which is inspired by the observation of a topological superconductor at about 10 GPa and 2.5 K in Sb_2_Se_3_^9^. In fact, Bi_2_Te_3_, Sb_2_Te_3_, and Bi_2_Se_3_ have been shown to be 3D topological insulators with insulating bulk electronic states and topologically protected metallic surface states^10,11^. The quest of new materials with topological insulating or superconducting properties by applying external pressure is one of the most promising topics in condensed matter science. Second, the A_2_B_3_ (A = Sb, Bi and B = S, Se, Te) chalcogenides exhibit exceptional thermoelectric properties, which can be drastically enhanced by application of external pressure^12–16^. Such a “tuning” feature of the physical properties may be attributed to a pressure-induced electronic topological transition (ETT). These studies of the properties of A_2_B_3_ materials under pressure could help in the design of better thermoelectric materials.

In the current work, in order to study the compression behaviors of Sb_2_S_3_ under high pressures and to clarify the high-pressure phase transition of Sb_2_S_3_, we have carried out a comprehensive investigation of Sb_2_S_3_ at room temperature by synchrotron XRD using a diamond anvil cell (DAC) up to 50.2 GPa. We have identified that Sb_2_S_3_ experiences a series of high-pressure (HP) induced phases (phase II at 12.6 GPa, phase III at 16.9 GPa and phase IV at 21.3 GPa) and eventually develops into a body-centered cubic disordered structure (phase V above 28.2 GPa). We report here the determination of the high-pressure *bcc* phase and the electronic topological evolution of Sb_2_S_3_. Possible candidates for the crystal structures of the intermediate phases (phase II, phase III and phase IV) are suggested, which are still under investigation.

## Experimental Details

Commercially available Sb_2_S_3_ powders (Alfa Aesar, 99.999%. The average particle size of the sample is about 2-3 *μ*m in diameter, and each particle contains a single crystalline grain, as shown in Fig. 2 in the supplementary materials.) were loaded into a hole of 50 µm in diameter drilled in a stainless steel gasket and compressed between two 300 *µ*m culets diamond anvils. Pressure was determined with the ruby fluorescence method^17^. A methanol-ethanol (4:1) mixture was used as the pressure transmitting medium. *In situ* high-pressure angle-dispersive X-ray diffraction (ADXRD) experiments were carried out at BL15U1 beamline of the Shanghai Synchrotron Radiation Facility (SSRF). The monochromatic beam wavelength used for data collection was 0.6199 Å with a focus spot of 2 × 3 *μ*m^2^ in size. The Bragg diffraction images were collected using MAR-165 charge coupled device (CCD) detector. The average acquisition time was 10 s. The two-dimensional XRD images were analyzed by using FIT2D software^18^, yielding one-dimensional intensity versus diffraction angle 2*θ* patterns^19^. The sample-detector distance and geometric parameters were calibrated using a CeO_2_ standard from NIST. High-pressure synchrotron XRD patterns were fitted by LeBail profile matching refinements by using the GSAS + EXPGUI programs^20,21^.

## Results and Discussion

**Figure 1:**
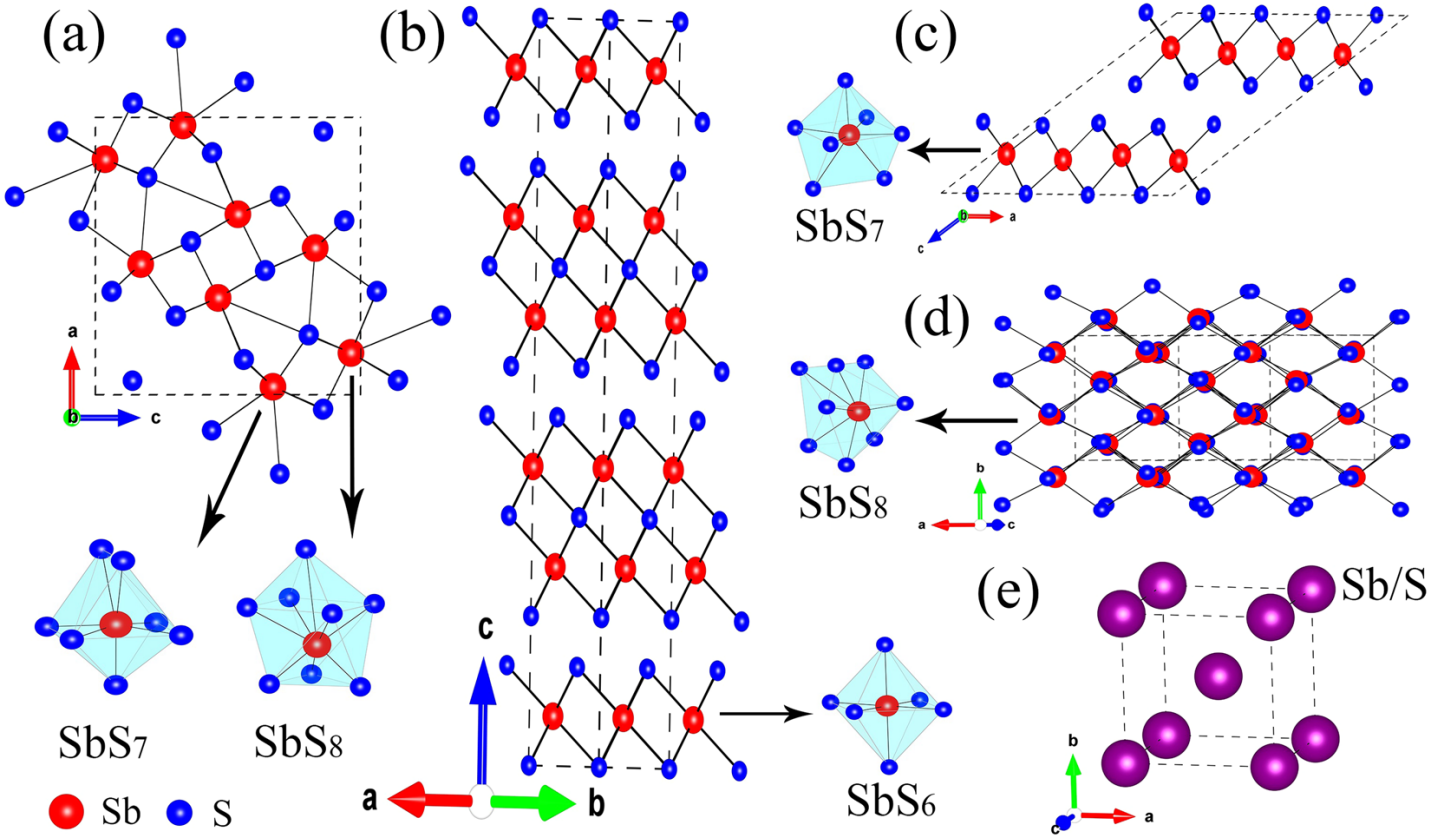
The sketch maps of the crystal structures of (**a**) *Pnma*, (**b**) *R-*3*m*, (**c**) *C2/m*, (**d**) *C2/c*, and (**e**) disordered *Im-*3*m* phases of Sb_2_S_3_. Large and small spheres represent Sb and S atoms, respectively.

**Figure 2:**
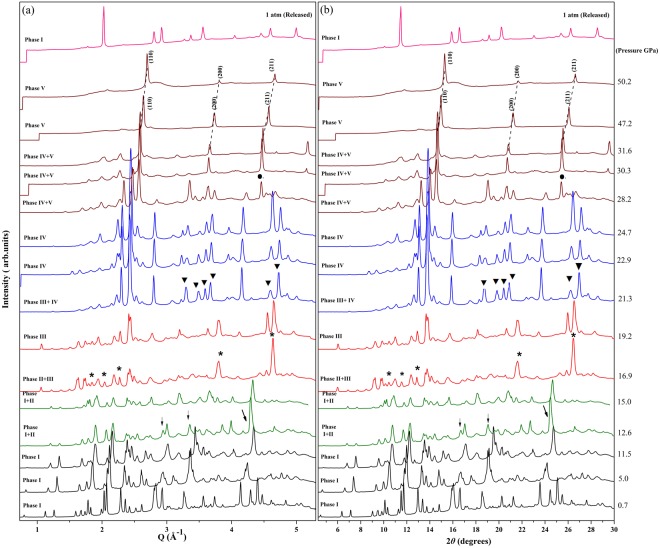
XRD patterns collected in (**a**) I(Q) style and (**b**) I(2θ) style at selected pressures for Sb_2_S_3_ with an incident wavelength λ = 0.6199 Å. The peaks marked as arrows, asterisks, solid triangles, and solid circles are the diffraction peaks for the emerging phases II, III, IV and V, respectively. The pattern at the top was observed at ambient pressure after releasing pressure.

**Figure 3:**
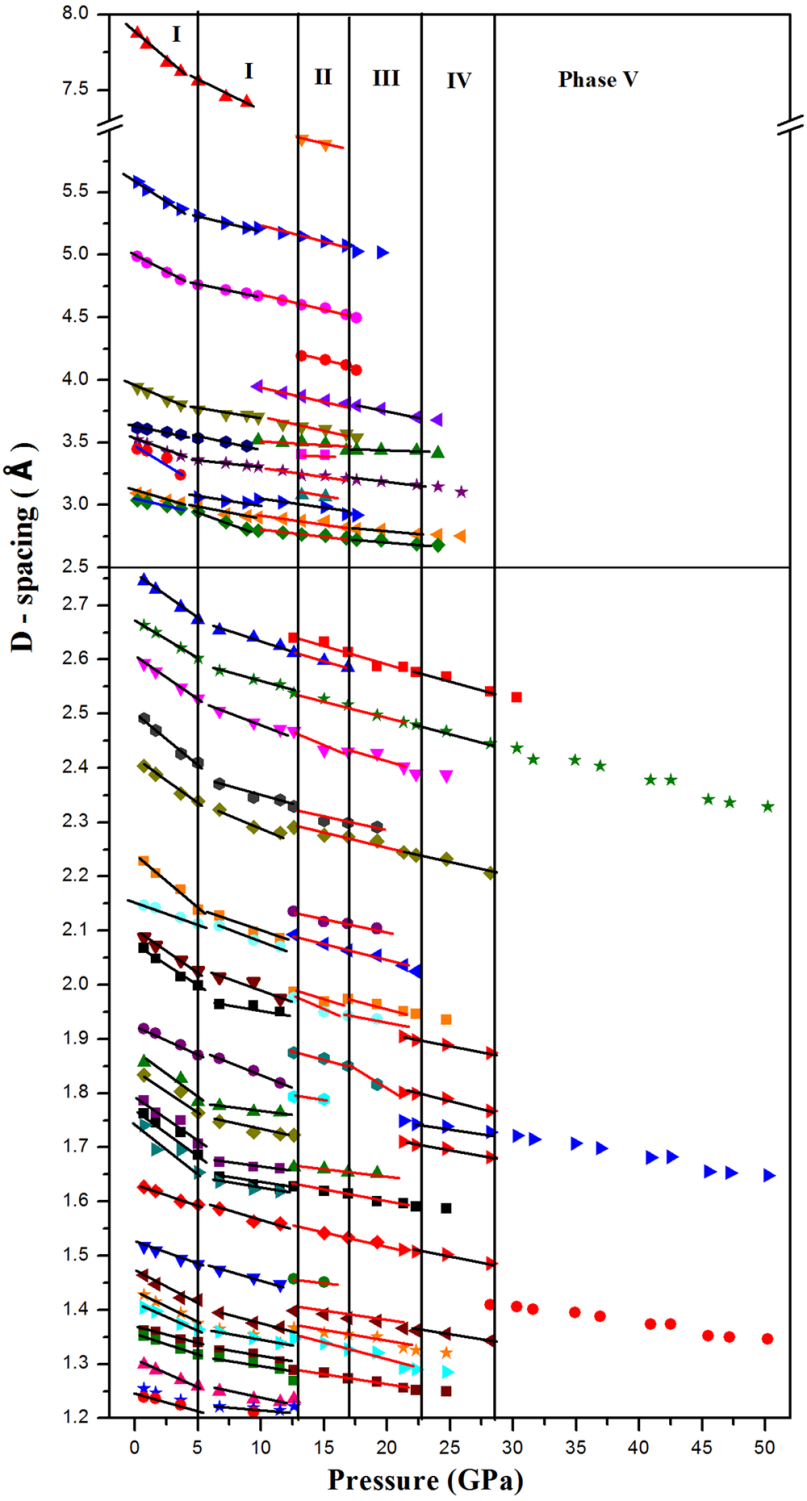
The pressure dependent lattice-spacings of Sb_2_S_3._ The existing regions of the identified phases are labeled in the figure. Around transition pressure, some slopes exhibit abrupt changes.

The crystal structures of Sb_2_S_3_ phases are schematically shown in Fig. 1. At ambient conditions, Sb_2_S_3_ adopts an orthorhombic structure (SG *Pnma*, Z = 4, U_2_S_3_-type). The Sb^3+^ ions are located at two different sites in this phase, and their coordination environments can be described as sevenfold for the Sb(1) site and eightfold (7 + 1) for the Sb(2) site, respectively (Figs 1(a) and 2) shows the selected X-ray diffraction patterns of Sb_2_S_3_ as a function of pressure up to 50.2 GPa. All the reflections obtained at 0.7 GPa can be indexed by space group *Pnma* with lattice parameters a = 11.303, b = 3.814, and c = 11.196 Å, via LeBail refinement using the GSAS software, which is in close agreement with earlier reported values^22^. Four structural phase transitions were clearly observed in the pressure range of 0–50.2 GPa, as seen in Fig. 2. With increasing pressure, the obvious changes may be distinguished in the patterns. New diffraction peaks start to appear in the XRD patterns at pressures above 12.6 GPa (marked by arrows in Fig. 2), indicating the onset of a structural phase transition (phase II). The reflections from phase III and phase IV are observed at pressures above 16.9 GPa (marked by asterisks in Fig. 2) and above 21.3 GPa (marked by triangles in Fig. 2), respectively. When the pressure increases up to 28.2 GPa, phase IV starts to transit into a new phase V. Above 42.5 GPa, the transformation is complete and no further transition is observed up to 50.2 GPa. The phase transitions are reversible. After a full pressure release, Sb_2_S_3_ was recovered to the ambient-pressure structure (see the pattern at the top of Fig. 2). The pressure dependences of the lattice-spacings show also the obvious trend of the phase transitions, as demonstrated in Fig. 3. These findings are considerably different from two recent high-pressure studies on Sb_2_S_3_^7,8^. A joint experimental and theoretical study of Sb_2_S_3_ under compression indicates that neither a first-order phase transition nor any second order isostructural phase transition has been observed up to 25 GPa^7^. On the contrary, two phase transitions have been observed in another combined high-pressure Raman and XRD investigation^8^. A second-order isostructural transition arising from changes in the electronic structure of Sb_2_S_3_ occurs at 5 GPa. The second transition at 15 GPa is characteristic of the onset of structural disorder, which is speculated to be a transient state of the possible new high-pressure phase^8^. Several factors may be taken into consideration as for the differences in the results by different groups. First, it is important to have in view the compositions, geometric shapes, sizes, and microstructures, of the samples employed. These features play an important role in the compression behaviors of materials under high pressures^23^. The second point concerns with the experimental details, such as the pressurizing procedures, the pressure intervals, the accelerating rate of pressures, and the pressure-maintaining period of time, etc. The coexistence of the two- or multi-phases indicate that the phase transformations of Sb_2_S_3_ are sluggish and respond slowly to the applied pressures, which make the experimental results very sensitive to pressurizing procedures.

**Figure 4:**
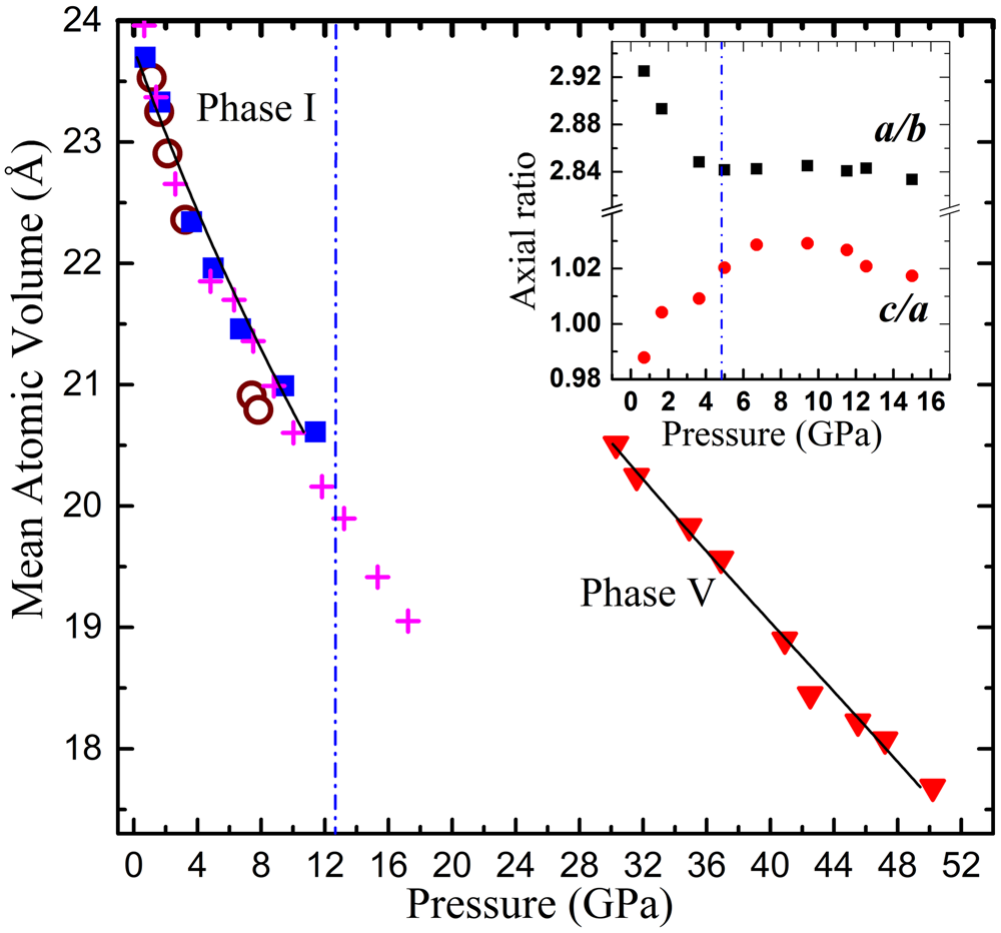
The diffraction profiles of the high-pressure phases of Sb_2_S_3_ at (**a**) 12.6 GPa, (**b**) 16.9 GPa, (**c**) 21.3 GPa and (**d**) 42.5 GPa, respectively. The solid lines and open circles represent the fits for the lattice and observed data, respectively, and the solid lines at the bottom are the residual intensities. The vertical bars indicate the peak positions. Diffraction patterns in (**a**), (**b**) and (**c**) are a mixture of phases II and I, phases III and II, phases IV and III, respectively.

The experimental XRD pattern of phase V is simple and neat, and can be indexed to a bcc unit cell (SG *Im-3m*, Z = 2) through Rietveld refinement [Fig. 4(d)]. This determination leads to a = 3.361 Å, allowing only one inequivalent atomic position at 2a (0, 0, 0). Within the *Im-3m* structure, Sb and S atoms are disordered and randomly share the bcc lattice sites, forming an Sb-S substitutional solid solution, similar to the situations in the high pressure induced substitutional solid solution phases in Bi_2_Te_3_, Sb_2_Te_3_ and Sb_2_Se_3_ compounds^1,3,4^. The structural parameters for the identified structures at selected pressures are listed in Table 1.

**Table 1:** Experimental lattice parameters and atomic coordinates of Sb_2_S_3_ at selected pressures.

Phase	Lattice parameters (Å)	Atom	Position	x	y	z	B_0_ (GPa)	B_0_′
*P nma*	a = 11.303	S1	4c	0.2152	0.25	0.8039	41.4	7.8
0.7 (GPa)	b = 3.814	S2	4c	0.3722	0.25	0.0607	37.6	3.8^a^
	c = 11.196	S3	4c	0.0514	0.25	0.1405	26.9	7.9^b^
	α = β = γ = 90°	Sb1	4c	0.3498	0.25	0.4633	27.2	6.0^c^
		Sb2	4c	0.0294	0.25	0.6703		
*R -3m*	a = 4.507	S1	6c					
12.6 (GPa)	b = 4.507	S2	6c					
	c = 30.776	Sb1	3a					
	γ = 120°							
*C2/m*	a = 14.968	S1	4i					
16.9 (GPa)	b = 3.985	S2	4i					
	c = 17.723	S3	4i					
	β = 149.729°	Sb1	4i					
		Sb2	4i					
*C2/c*	a = 10.194	S1	8 f					
21.3 (GPa)	b = 6.817	S2	8 f					
	c = 10.543	Sb1	4e					
	β = 136.985°							
*Im-3m*	a = 3.362	Sb/S	2a	0	0	0	74.6	2.5
47.2 (GPa)	α = β = γ = 90°							

^a^ref.^7^.

^b^ref.^22^.

^c^ref.^8^.

It is noteworthy that weak and broad humps exist in the patterns at high pressures. The humps can be very misleading, since they are generally assigned to amorphization in high pressure XRD experiments. However, in our case, the possibility of them originating from amorphization may be ruled out, since the simultaneous transitions into the crystalline bcc phase and the amorphous states are quite unlikely. In fact, the humps may well be attributed to the diffuse scattering instead of amorphization, as was evidenced in the *in situ* angle dispersive x-ray diffraction studies on elemental Zr and Ti, which belies the amorphous formation^24^.

**Figure 5:**
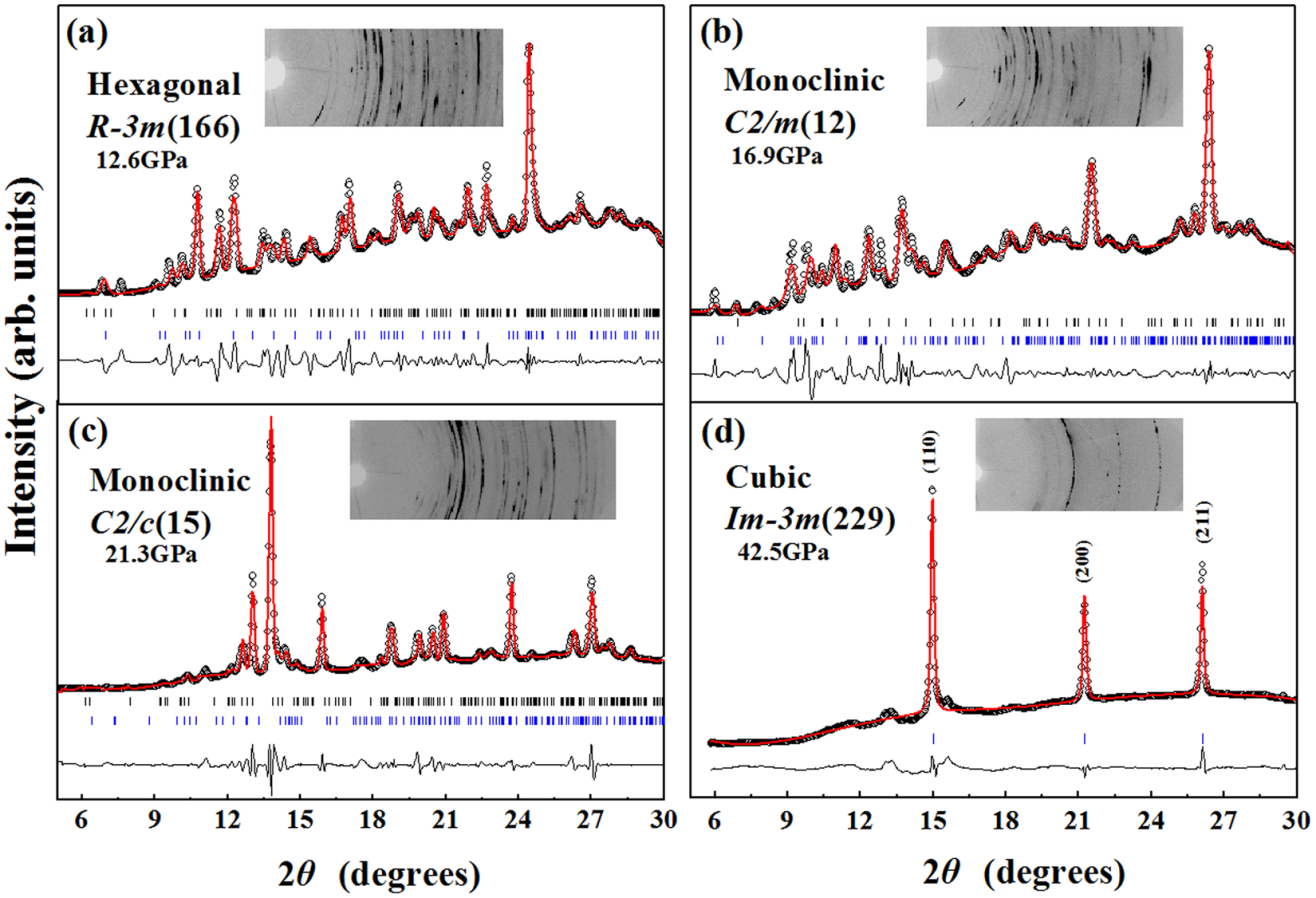
Pressure dependence of the atomic volume for phases I and V. Closed triangle indicate the atomic volume of phase V. The atomic volume of phase I is indicated by closed squares, open circles and cross symbols, corresponding to data from the present data, Lundegaard *et al*.^22^ and Efthimiopoulos *et al*.^8^, respectively.

Figure 5 shows the pressure dependence of the volume per atom in phase V. The volume per atom continuously decreases with increasing pressure. The pressure- induced substitutional solid solution is unprecedented in the sulfide family of these group VA elements, as mentioned in the introduction section. This work represents a significant step forward in understanding the HP structural evolution of the A_2_B_3_ (A = Sb, Bi; B = Se, Te) series, and also provides a novel way to the search for new types of solid solutions. The fitting of the *P-V* data for phase V to the third-order Birch-Murnaghan Equation of State yielded the bulk modulus *B*_0_ = 74.6 GPa with the first derivatives *B′*_0_ = 2.5.

It is amazing that the system exhibits a seemingly anomalous response to pressure, i.e., the high-pressure phase V have a volume per atom comparable to or even higher than that of the low-pressure initial phases, as shown in Fig. 5. Such a flat response to pressure may be interpreted in terms of microstructure effects of the multi-phase coexisting fields. Upon pressurizing, the phase transformation occurs, resulting in basket-weave microstructures within the grains, due to the coexistence of the phases. The interwoven stresses among the coexisting phases build up an additional potential acting against the applied pressure. Thus the atomic volume of each phase is less compressed than it would be in the single-phase situation. The phase transformation is sluggish and proceeds gradually. The system responds to phase transition rather than atomic volume compression. The volume increasing phase transformation competes with the volume compression in each phase, resulting in the flat response to pressure. Similar phenomena and effects have been already reported for the pressure-induced phase transformations with two- or multi-phase coexistence, such as in *γ*-based titanium aluminide intermetallics^25^.

The pressure dependence of the observed c/a ratio (for phase I) were also shown in the inset of Fig. 5. It can be seen a maximum in the c/a ratio appears at around 5 GPa, which suggest the presence of an ETT in this compound near 5 GPa. The change of the c/a ratio with pressure is a good indicator of the ETT, since it is caused by the anomalous behavior of *a* as a function of pressure, which in turn results in a change in the compressibility at the ETT^26^. The observed pressure dependences of the lattice spacings exhibit abrupt changes in their slopes at around 5 GPa (see Fig. 3). These phenomena in combination indicate that the elastic properties of Sb_2_S_3_ undergo considerable changes, concomitant with the ETT occurring at 5 GPa. An ETT occurs when an extreme of the electronic band structure, which is associated with a Van Hove singularity in the density of states, crosses the Fermi energy (EF), and leads to a strong redistribution of the electronic density of states (EDOS) near EF^27^. High-pressure electrical transport measurements corroborated the presence of isostructural ETT in Sb_2_S_3_^24^. The existence of ETT by the phonon spectrum measurements have also been observed in the compounds of the A_2_B_3_ chalcogenide series, such as Sb_2_S_3_, Sb_2_Te_3_, Bi_2_Te_3_, and Bi_2_Se_3_^26–29^.

The formation mechanism of the bcc solid solutions of these A_2_X_3_ (A = Sb, Bi and X = Se, Te) chalcogenides are generally explained in terms of atomic size and electronegativity. With increasing pressure, the atomic radii in these chalcogenides become approximately equal, making it possible for them to form substitutional solid solutions at high pressures (Hume−Rothery rules^30^). On the other hand, according to the Pauling scale, the compounds comprised of these group VA and VIA elements with similar electronegativity values are favorable to form substitutional solid solutions. It is reported that the ambient-pressure *Pnma* structure of Bi_2_S_3_ is found to persist up to 50 GPa through a combination of experimental and theoretical studies^6^. Further compression leads to structural disorder and amorphization. Interestingly, for Sb_2_Se_3_, which adopts the same initial structure as Sb_2_S_3_, the initial *Pnma* phase of Sb_2_Se_3_ transform directly into the disordered bcc structure (SG *Im-3m*, Z = 2) above 51 GPa without any intermediate phase^4^. The different structural sequences between Sb_2_S_3_ and Sb_2_Se_3_ under pressure may be caused by atomic radii and electronegativity, since S has a similar electronegativity value as Se (2.58 for S and 2.55 for Se) but a much smaller atomic size (1.09 Å for S and 1.22 Å for Se). A direct structural transition from the *Pnma* phase into any other structure type has not previously been observed for any A_2_B_3_ materials.

The unambiguous assignment of the other observed high pressure phases is difficult, since the diffraction peaks of them coexist in the XRD patterns. However, through comparison of the high pressure behaviors within A_2_B_3_ family, useful hints can be acquired, which may help to resolve the phase sequences of Sb_2_S_3_ under high pressures. Our previous simulations and XRD experiments unraveled that the heavier Bi_2_Te_3_ and Sb_2_Te_3_ compounds with the initial *R-3m* phase first transform into low HP phases II and III, which adopt monoclinic sevenfold *C*2/m and eightfold *C*2/c structures, respectively, and then into bcc structure (*Im-3m*, Z = 2). From this viewpoint, we speculate that the pressure-induced structural phase transition sequence of Sb_2_S_3_ may be *Pnma* → *R-*3*m* → *C2/m* → *C2/c* → disordered *Im-*3*m*. Figure 4 shows the LeBail refinements for the XRD patterns of Sb_2_S_3_ at 12.6, 16.9 and 21.3 GPa based on the above speculation. It can be seen that the overall consistencies of the simulated patterns with the experimental data are acceptable, indicating our speculation is reasonable.

## Conclusions

In summary, we have performed compression behavior studies of Sb_2_S_3_ by using advanced *in situ* angle-dispersive synchrotron X-ray diffraction techniques and clarified the high-pressure induced phase transitions of Sb_2_S_3_. We have experimentally demonstrated that Sb_2_S_3_ undergoes four different HP structural phase transitions upon compression to 50.2 GPa. The crystal structure transforms ultimately to the high-symmetry cubic phase at pressures above 28.2 GPa, forming an Sb-S substitutional solid solution, similar to those observed in Bi_2_Te_3_, Sb_2_Te_3_ and Sb_2_Se_3_ under high pressures. The solid solution adopts a bcc disordered structure stable at pressures >28.2 GPa and up to at least 50.2 GPa.

## Electronic supplementary material

Supplementary Materials
