# Supplementary material for: Experimental Observation of the High Pressure Induced Substitutional Solid Solution and Phase Transformation in Sb2S3
Source: Sci Rep. 2018 Oct 4;8:14795. doi: 10.1038/s41598-018-33035-4 (PMC6172247; doi:10.1038/s41598-018-33035-4)
Supplement: Supplementary file 1 — Supplementary Materials [file 41598_2018_33035_MOESM1_ESM.docx]

**Experimental Observation of the High Pressure Induced Substitutional Solid Solution and Phase Transformation in Sb_2_S_3_**

Yingying Wang^1^, Yanmei Ma^1＊^, Guangtao Liu^2^, Jianyun Wang^1^, Yue Li^1^, Quan Li^1^, Jian Zhang^1＊^, YanmingMa^1^, Guangtian Zou^1^

^1^College of Physics, State Key Laboratory of Superhard Materials, Jilin University, Changchun 130012, China

^2^National Key Laboratory of Shock Wave and Detonation Physics, Institute of Fluid Physics, Chinese Academy of Engineering Physics, Mianyang 621900, China

**Supplementary Materials**


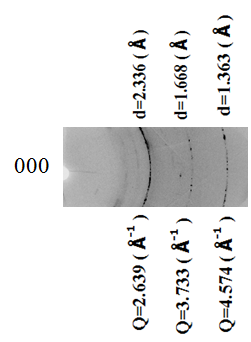


**Supplementary Fig.1**. two-dimensional X-ray diffraction images of Sb_2_S_3_ at pressure of 42.5 GPa and ambient temperatures obtained in an angle-dispersive mode. Each Q(d) value corresponds to a diffraction ring. The scale for Q and d-spacing are given, and they correspond to each other.


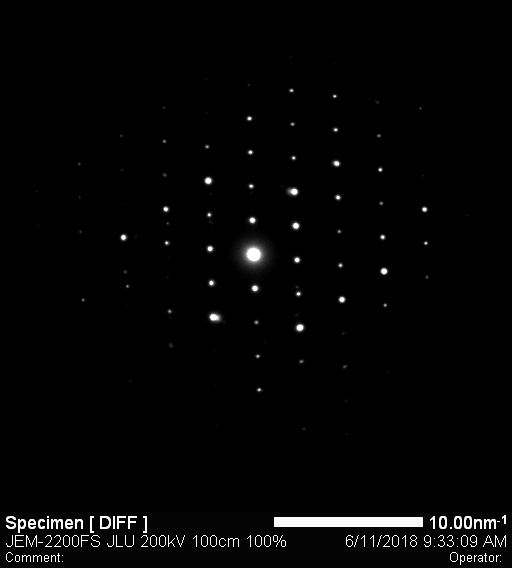

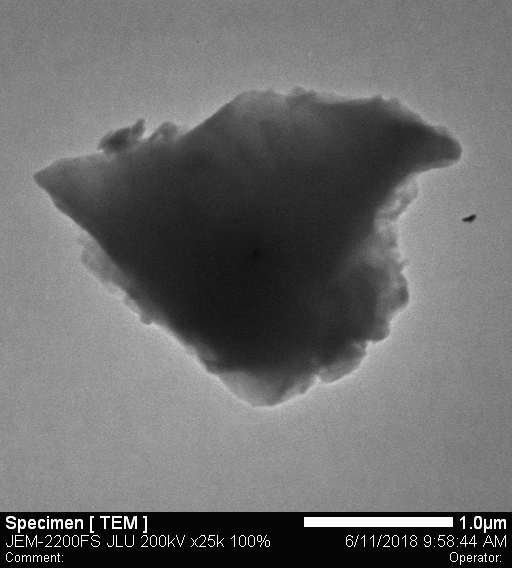

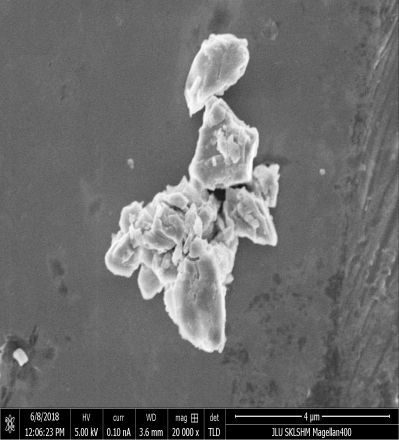


(a) (b) (c)

**Supplementary Fig.2**. The morphological studies of the sample powders via SEM (a) and TEM (b,c) techniques. The select area electron beam diffractions (SAED) indicate each particle is a single crystalline grain. The particle size of the sample is about 2-3 μm in diameter.


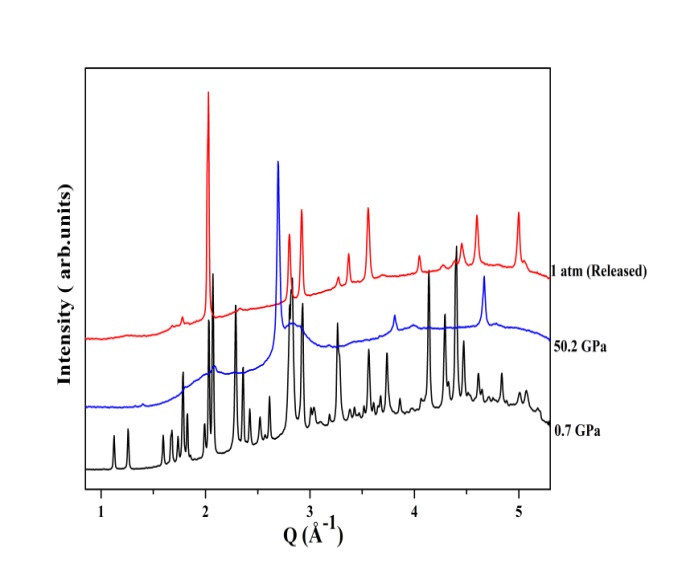

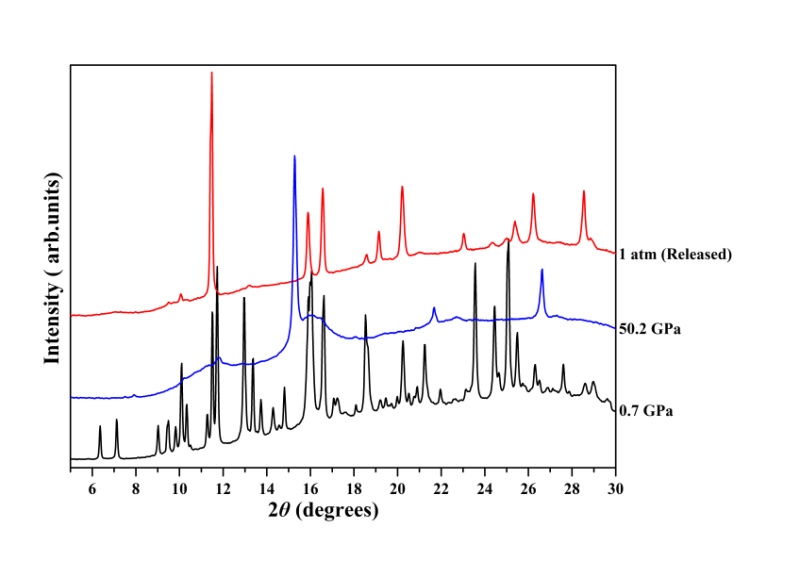


**Supplementary Fig.3**. XRD patterns collected at 0.7GPa, 50.2 GPa and after pressure release for Sb_2_S_3_ with an incident wavelength λ = 0.6199 Å. The existing peaks in the released pattern match those in the starting pattern very well. Apparently, there is not *bcc* phase quenched
